# A Case of Removal of the Major Portion of the Lower Jaw for Sarcoma

**Published:** 1896-07

**Authors:** Thomas S. K. Morton

**Affiliations:** Professor of Surgery in the Philadelphia Polyclinic, etc.


ARTICLE VI.

### A CASE OF REMOVAL OF THE MAJOR PORTION OF THE LOWER JAW FOR SARCOMA.

BY THOMAS S. K. MORTON, M. D.

Professor of Surgery in the Philadelphia Polyclinic, etc.

A colored man aged 21 years presented himself, November 1, 1895, at the Polyclinic Hospital, for treatment of a large swelling upon the left slde of his lower jaw. The disease, he stated, originated in a decayed tooth-root (probably bicuspid) and had been noticeable as a growing tumor for about eighteen months. During this time two molar teeth had been forced out by the growth, and he had lost much flesh and strength. Pain in the affected parts had not been marked. The act of chewing had become impossible, and even swallowing was difficult. A superficial operation (probably simply curettage)

had been performed by another surgeon soon after the vegetative process first made its appearance. No relief followed and rapid growth continued. Upon examination the left side of the man's face was found to be much distorted and appeared as if a small orange rested between the jaw and the cheek. Upon separating the jaws it was observed that a mass the size of a hen's egg projected from the inner left side of the mannible toward the right, in an upward direction, displacing the tongue before it and largely filling the mouth. Pulling the cheek outward the growth was seen to involve the whole body of the left side of the jaw as well as the alveolus and contiguous mucous membrane. The skin was not infiltrated. The remaining left lower bicuspid as well as the canine and one incisor, together with the alveolar process containing their roots, were displaced as a whole, outward and downward, by growth of the tumor beneath. The mass was red, hard, very vascular, not sensitive, and was ulcerated upon its buccal surface. No hemorrhage has taken place. Neither the tongue, the mouth, nor neighboring glands appeared to be infiltrated.

Diagnosis of sarcoma was made. Excision of the jaw from the first *right* molar to and including the left condyl was advised and accepted. It was considered best to go considerably beyond the middle line of the jaw in removing it so as to diminish as much as possible the chances of local recurrence.

Ether was administered and an incision made beginning one half inch below the lip at the site of the first right bicuspid tooth and carried vertically down to the bone and well around the lower margin of the jaw. The lip was purposely not divided anywhere during the operation, because sufficient room for excision of the lower jaw can generally be secured without severing it, and, moreover, the cosmetic effect, which is never too good after this operation on account of the asymmetry of face resulting, is greatly enhanced by an uninjured lip line. A second incision was next carried from the termination of the first, laterally to the left, one and a half inch behind and below the margin of the jaw to its angle. The direction of the incision was then changed so that it ran upwards along the edge of the ramus to the level of the lobe of the ear, where it was terminated so as to avoid cutting branches of the facial nerve. This extensive cut, throughout its entire extent, except where the facial artery was crossed, was carried down to, but not through, the periosteum. The space where the facial artery passed over the jay was next carefully dissected, the vessel cleared, and then ligated. By thus dealing with that artery both blood and annoyance are saved. The muscular attachments to the outer and under surfaces of the jaw were next everywhere separated from the periosteum by blunt dissection, until the bone was cleanly stripped in these regions. The periosteum in malignant cases should never be allowed to remain because of the great danger of recurrence of growth in it. Hence in the present instance it was sacrificed with and to the full extent of the removed bone. When the mucous membrane of the mouth was come upon it was divided as far away from the tumor, out upon the cheek, as possible.

Next the first right bicuspid root was extracted and the jaw divided by a narrow saw at that point. The two portions of the jaw were now pried apart and a hook introduced into the portion to be removed, whereby it was forcibly drawn outwards. As it was thus held, the muscular attachments of the tongue and neck muscles, as well as the mucous membrane lining of the mouth, were rapidly clipped away from it with blunt-pointed scissors. By first dividing the jaw and separating its attachments while they were put upon the stretch, as the portion of the bone to be removed is drawn outwards, the procedure is rendered easier, safer and more precise.

The inferior dental artery at this stage was isolated at a point just above where it enters the inferior dental foramen, ligated, and, with its accompanying nerve, divided. The jaw was then drawn strongly downwards so as to bring the coronoid process into view and make tense the tendon of the temporal muscle which was divided by blunt scissors just above its insertion. The attachments of the pterygoids were next bluntly dissected from the ramus, the capsule of the temporo-maxillary joint incised, and the jaw readily removed upon severance of a few remaining posterior bands of the capsule. The tongue during these latter manipulations was controlled by a silk thread passed through its tip and held where desired by an assistant. Now the muscles beneath the tongue were firmly united by sutures to the fibrous tissues outside of the sawn edge of the remaining portion of jaw. This at once gave the patient full control of the member and ability to swallow as soon as he recovered from the anesthetic. Not for a day later, however, was it thought safe to remove the sling-thread. All suspicious mucous membrane having been dissected away, the mucous margins of the wound were accurately sutured, thus cutting off all raw surfaces from communication with the mouth. The skin was then separately stitched, leaving a little acetanilid gauze packing running from the posterior angle of the incision up into the cavity of the former temporo-maxillary articulation. No shock resulted, and the patient reacted well. Exceedingly little blood was lost.

The sutured mucous membrane held tight for two days before leaking. Then partial infection took place. Prompt and satisfactory recovery, however, ensued, and the patient was able to return to his home in another state in three weeks.

Microscopic examination demonstrated the growth to be a large spindle-celled sarcoma.

Reports from him six months later were very favorable as to comfort, facility in eating with remaining portion of jaw, non-recurrence of tumor, and gain of flesh and strength.—*The Philadelphia Polyclinic.*

